# Selenium and the risk of cancer of the lung and larynx. A case-control study from a region with low selenium

**DOI:** 10.1186/1897-4287-10-S3-A7

**Published:** 2012-04-20

**Authors:** Katrzyna Jaworska, Satish Gupta, Katarzyna Durda, Magdalena Muszynska, Grzegorz Sukiennicki, Elżbieta Jaworowska, Tomasz Grodzki, Mieczysław Sulikowski, Piotr Woloszczyk, Janusz Wójcik, Jakub Lubiński, Cezary Cybulski, Tadeusz Dębniak, Marcin Lener, Steven A Narod, Ping Sun, Jan Lubiński, Anna Jakubowska

**Affiliations:** 1 International Hereditary Cancer Centre, Department of Genetics and Pathology, Pomeranian Medical University, Szczecin, Poland; 2 Postgraduate School of Molecular Medicine, Warsaw Medical University, Warsaw, Poland; 3 Department of Otolaryngology and Laryngological Oncology, Pomeranian Medical University, Szczecin, Poland; 4 Department of General Thoracic Surgery, Pomeranian Medical University, Szczecin, Poland; 5 Maxillofacial Surgery, Pomeranian Medical University, Szczecin, Poland; 6 Department of Toxicology and Molecular Pathobiochemistry, Pomeranian Medical University, Szczecin, Poland; 7 Women’s College Research Institute, Toronto, ON, Canada

## 

Selenium deficiency has been suggested by several studies to be associated with cancer risk. We conducted a case-control study in Szczecin, a region of northwestern Poland, on 86 cases of lung cancer, 87 cases of laryngeal cancer and an equal number of healthy controls. We studied the serum level of selenium and genotypes for four variants in four selenoprotein genes (GPX1, GPX4, TXNRD2 and SEP15) and the odds of being diagnosed with lung or laryngeal cancer.

Among lung cancer cases, the mean selenium level was 63.2 µg/l, compared to a mean level of 74.7 µg/l for their matched controls (p < 0.0001). Among laryngeal cancer cases, the mean selenium level was 64.8 µg/l, compared to a mean level of 76.3 µg/l for their matched controls (p < 0.0001). Compared to a serum selenium value in the lowest of four categories (≤ 60 µg/l) a selenium level in the highest category (> 80 µg/l) was associated with an odds ratio of 0.10 (95% CI 0.03 to 0.34; p = 0.0002) for lung cancer and 0.24 (95% CI 0.10 to 0.59; p = 0.002) for laryngeal cancer. In four selenoproteins studied here we found a modest associations of genetic variants in GPX1 and GPX4 with lung and TXNRD2 with laryngeal cancer risk.

In this region of endemic low selenium level, there is a strong inverse association between the level of serum selenium and the risks of lung and laryngeal cancer.

